# Supplementary material for: Inactivation of Ppp1r15a minimises weight gain and insulin resistance during caloric excess in female mice
Source: Sci Rep. 2019 Feb 27;9:2903. doi: 10.1038/s41598-019-39562-y (PMC6393541; doi:10.1038/s41598-019-39562-y)
Supplement: Supplementary file 1 — Supplementary Figure S1 [file 41598_2019_39562_MOESM1_ESM.pdf]

Supplementary Figure S1

Inactivation of *Ppp1r15a* minimises weight gain and insulin resistance during caloric excess in female mice

Vruti Patel, Guillaume Bidault, Joseph E. Chambers, Stefania Carobbio, Angharad J. T. Everden, Concepción Garcés, Lucy E. Dalton, Fiona M. Gribble, Antonio Vidal-Puig and Stefan J. Marciniak

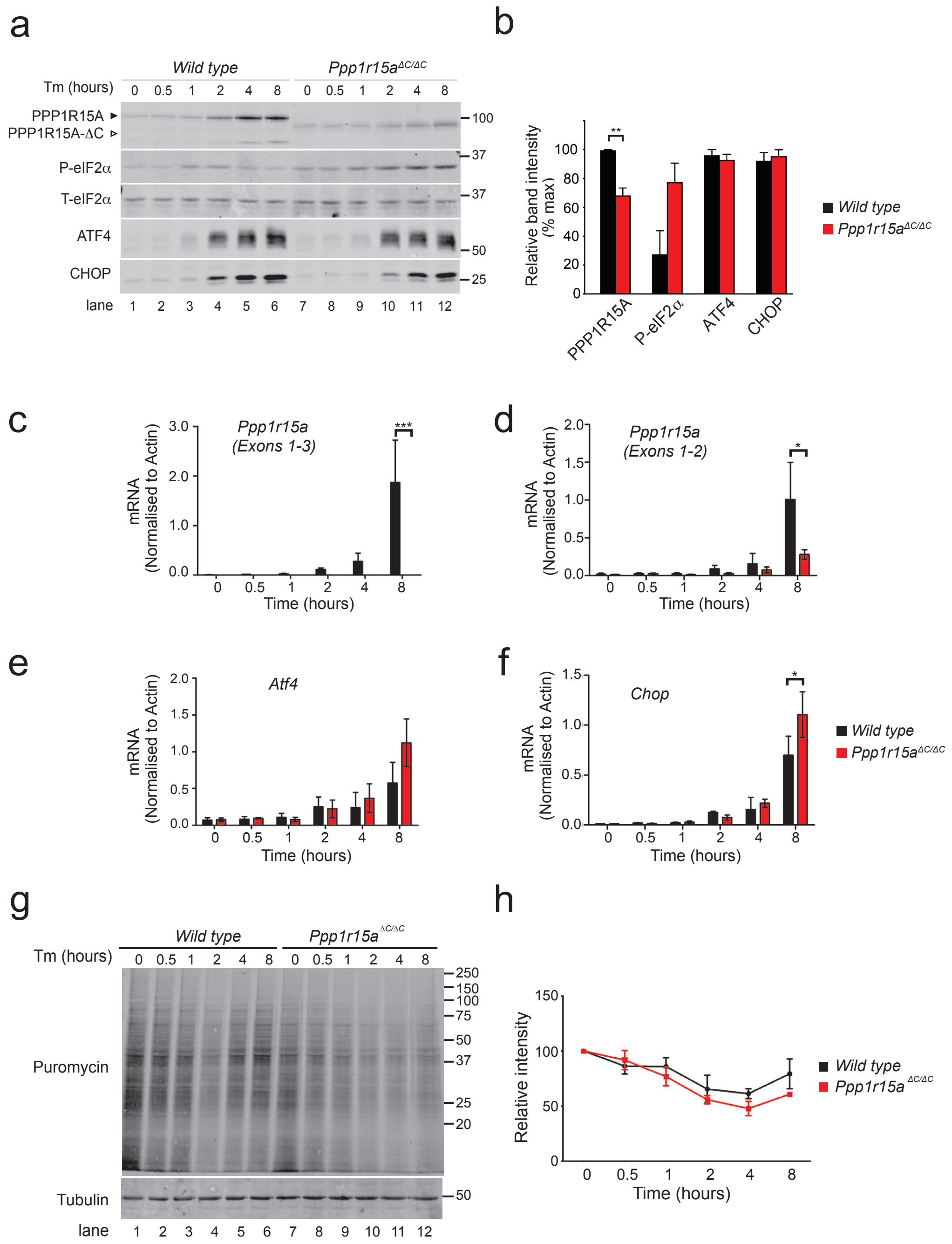

Supplementary Figure 1. Inactivation of PPP1R15A reduces ER stress in response to tunicamycin

(a) Immunoblot for PPP1R15A, P-eIF2α, T-eIF2α, ATF4 and CHOP in lysates of wild type and Ppp1r15aΔC/ΔC MEFs following treatment with tunicamycin (Tm) 2µg/mL for indicated times. Proteins of the expected sizes are marked with a solid triangle for PPP1R15A or an open triangle for PPP1R15A-ΔC. Molecular size markers shown in kDa.

(b) Quantification of (a) using ImageJ software.

(c-f) Wild type and Ppp1r15aΔC/ΔC MEFs were treated with tunicamycin 2µg/mL for indicated times and RNA was prepared. Ppp1r15a (Exons 1-3), Ppp1r15a (Exons 1-2), Atf4, and Chop were quantified relative to actb by qRT-PCR. N=3; mean ± SEM. P value calculated by two-way ANOVA.

(g) Immnoblot for puromycin and tubulin in lysates of wild type or Ppp1r15aΔC/ΔC MEFs following treatment with tunicamycin (Tm) 2µg/mL for indicated times. Ten minutes prior to harvesting, puromycin was added to the culture medium at a final concentration of 10ng/mL. Molecular size markers shown in kDa.

(h) Immunoreactivity to puromycin within lysates served as a marker of protein translation and was quantified using ImageJ software. *** $p<0.001$, ** $p<0.01$, * $p<0.05$.
