# Supplementary material for: Inactivation of Ppp1r15a minimises weight gain and insulin resistance during caloric excess in female mice
Source: Sci Rep. 2019 Feb 27;9:2903. doi: 10.1038/s41598-019-39562-y (PMC6393541; doi:10.1038/s41598-019-39562-y)
Supplement: Supplementary file 2 — Supplementary Table S1 [file 41598_2019_39562_MOESM2_ESM.pdf]

Supplementary Table S1

**Primer**	**Forward (5' – 3')**	**Reverse (5' – 3')**
mAtf4	GCAAGGAGGATGCCTTTTC	GGTTCCAGGTCATCCATTCG
mPpp1r15a (Exons 1-2)	AGGGACGCCCACAACTTCTA	CAGGGGTGCTGGGTTTGTAT
mPpp1r15a (Exons 1-3)	CCCGAGATTCCTCTAAAAGCTC	CCAGACAGCAAGGAAATGG
mGrp78	GACTGCTGAGGCGTATTTGG	AGCATCTTTGGTTGCTTGTCG
mChop	GGAGCTGGAAGCCTGGTATGAG	GCAGGGTCAAGAGTAGTGAAGG
mActin	TCCTGGCCTCACTGTCCA	GTCCGCCTAGAAGCACTTGC
mPparγ	CAAGGCGAGGGCGATCTTGA	CGGATCGAAACTGGCACCCT
mScd1	TGCTGGGGCGAGACTTTTGT	TCCATAGAGATGCGCGGCAC
mFasN	GCACACACAATGGACCCCCA	CCCAGACGCCAGTGTTCGTT
mAcacα	TGGCGTCCGCTCTGTGATAG	TGTGCTGGGTCATGTGGACG
mCebpβ	AGCACAAGGTGCTGGAGCTG	ACAAGTTCCGCAGGGTGCTG
mPck1	CGAGACTAGCGATGGGGGTG	TGGCAGGGGTGCAGAATCTC
mIns1	GACCAGCTATAATCAGAGACC	AGTTGCAGTAGTTCTCCAGCTG
mIns2	AGCCCTAAGTGATCCGCTACAA	AGTTGCAGTAGTTCTCCAGCTG
mUcp1	CCCGCTGGACACTGCC	ACCTAATGGTACTGGAAGCCTGG
mPgc1α	AACCACACCCACAGGATCAGA	CTCTTCGCTTTATTGCTCCATGA
mDio2	TGCGCTGTGTCTGGAACAG	CTGGAATTGGGAGCATCTTCA
mElovl3	AAGGTTGTTGAACTGGGACGAC	GTGGTGGTACCAGTGGACAAA
